# Influence of Absorbable Calcium Sulfate-Based Bone Substitute Materials on Human Haemostasis—In Vitro Biological Behavior of Antibiotic Loaded Implants

**DOI:** 10.3390/ma11060935

**Published:** 2018-06-01

**Authors:** Dominik Pförringer, Norbert Harrasser, Marc Beirer, Moritz Crönlein, Axel Stemberger, Martijn van Griensven, Martin Lucke, Rainer Burgkart, Andreas Obermeier

**Affiliations:** 1 Klinikum rechts der Isar der Technischen Universität München, Klinik und Poliklinik für Unfallchirurgie, Ismaninger Str. 22, 81675 München, Germany; Marc.Beirer@mri.tum.de (M.B.); Moritz.Croenlein@mri.tum.de (M.C.); Martijn.vanGriensven@tum.de (M.v.G.); 2 Klinikum rechts der Isar der Technischen Universität München, Klinik für Orthopädie und Sportorthopädie, Ismaninger Str. 22, 81675 München, Germany; norbert.harrasser@mri.tum.de (N.H.); axel.stemberger@lrz.tum.de (A.S.); burgkart@tum.de (R.B.); aobermeier@tum.de (A.O.); 3 Chirurgisches Klinikum München Süd, Am Isarkanal 30, 81379 München, Germany; martin.lucke@artemed.de

**Keywords:** calcium sulfate formulations, calcium carbonate, tripalmitin, coagulation, in vitro, Herafill^®^-G, Osteoset^®^, gentamicin, vancomycin, tobramycin, FXIIa, C3a, F_1+2_

## Abstract

Calcium sulfate (CS) formulations are frequently implanted as antibiotically impregnated bone substitutes in orthopedic and trauma surgery to prevent or treat bone infections. Calcium ions have been discussed as candidates to accelerate blood coagulation. The goal of this study is to evaluate substance-specific influences of CS formulations on blood coagulation. Specific ELISAs were conducted to determine markers of activated blood coagulation after incubation of human blood with CS beads. Additionally, wettability with freshly drawn human blood was measured. Three different types of CS bone substitute beads were compared (CS dihydrate with tripalmitin, containing Gentamicin (Herafill^®^-G: Group A) or Vancomycin (CaSO_4_-V: Group B); and a CS hemihydrate with Tobramycin (Osteoset^®^: Group C)). Examinations were performed by ELISA assays for F_1+2_, FXIIa and C3a. Our results prove that none of the CS preparations accelerated single specific assays for activated coagulation markers. This allows the conclusion that neither Herafill^®^-G (CaSO_4_-G) nor CaSO_4_-V alter haemostasis negatively. Blood samples incubated with Osteoset^®^ display an elevated F_1+2_-activity. The addition of tripalmitin in Herafill^®^-G shifts the original into a significantly hydrophobic formulation. This was additionally proven by contact angle examination of the three substances with freshly drawn human blood, showing that acceleration of plasmatic coagulation is hindered by lipids and induced by surface effects caused by presence of rapidly soluble calcium ions in the Osteoset^®^ preparation.

## 1. Introduction

Infected bone defects are frequently addressed through implantation of various bone grafts, often based on calcium sulfate preparations containing antibiotics. The clinical side effects of such formulations are highly relevant to usability, as well as to potential risks and opportunities in defect healing situations.

In this study, two formulations which are commercially available in Germany, as well as a third, prospective formulation containing vancomycin to address MRSA-infections, were compared, regarding their specific effects on blood coagulation after implantation [1]. The blood coagulation cascade requires calcium ions, and efficiently acts on the activation of platelets [2]. Three antibiotically loaded [3] calcium-based formulations were compared for this experiment: Calcium Sulfate (CS) dihydrate with tripalmitin, containing gentamicin (Herafill^®^-G: Group A) and vancomycin (CaSO_4_-V: Group B); CS hemihydrate containing tobramycin (Osteoset^®^: Group C). CS is an inexpensive material known for its high biocompatibility [4], and functions as a carrier material [5,6] by incorporation of therapeutic substances. Calcium preparations are known to influence blood coagulation, rendering it meaningful to compare the substances’ effects on human blood coagulation. Markers of activated coagulation and of the complement system (also a serin protease system) were determined using specific ELISA assays. Furthermore, a comparison of the wettability of the substances using contact angle measurements allows conclusions to be drawn regarding superficial material-blood interaction.

## 2. Materials and Methods

For this controlled clinical trial, resorbable bone substitute materials consisting of CS dihydrate, gentamicin and tripalmitin (Herafill^®^-G, Heraeus, Wehrheim, Germany), CS dihydrate, vancomycin, and tripalmitin, as well as commercially available CS hemihydrate with tobramycin (Osteoset^®^, Wright Medical Group Inc., Memphis, Tennessee, USA), were compared. The beads are composed as follows: Herafill^®^-G has a 6.0 mm diameter, at 250 mg weight per unit, consisting of calcium sulfate dihydrate (71.6%), calcium carbonate (17.9%), tripalmitin (8.8%), and gentamicin sulfate (1.7%). The second group has a 3.0 mm diameter, at 35 mg per unit, consisting of calcium sulfate dihydrate (72.0%), calcium carbonate (18.0%), tripalmitin (8.9%), and vancomycin hydrochloride (1.1%). Osteoset^®^ has a 4.8 mm diameter, with 107.5 mg weight per unit, consisting of a hemihydrate modification of calcium sulfate (96.0%) and tobramycin sulfate (4.0%). The composition of bead implants is given in contents per weight.

For the ELISA assays, all three substances were compared. For the contact angle measurement a reduced comparison between Herafill^®^-G and Osteoset^®^ as well as a glass control group was conducted.

To understand the influence of calcium carriers on blood coagulation, samples were tested using ELISA assays for quantitative detection of relevant coagulation factors in 16 samples of freshly drawn human blood after contact with the bone substitute materials. The blood was drawn using a regular polyethylene syringe without addition of any anticoagulation substances. Prior to blood drawing, two sets of seven 15 mL falcon tubes were equipped with one bead and one additional pure blood control sample. Subsequently, they were filled with 4 mL fresh, untreated human blood, and incubated for 8 min. Incubation time was chosen in accordance with previous literature by Obermeier et al. 2012 [7].

**Figure 1:**
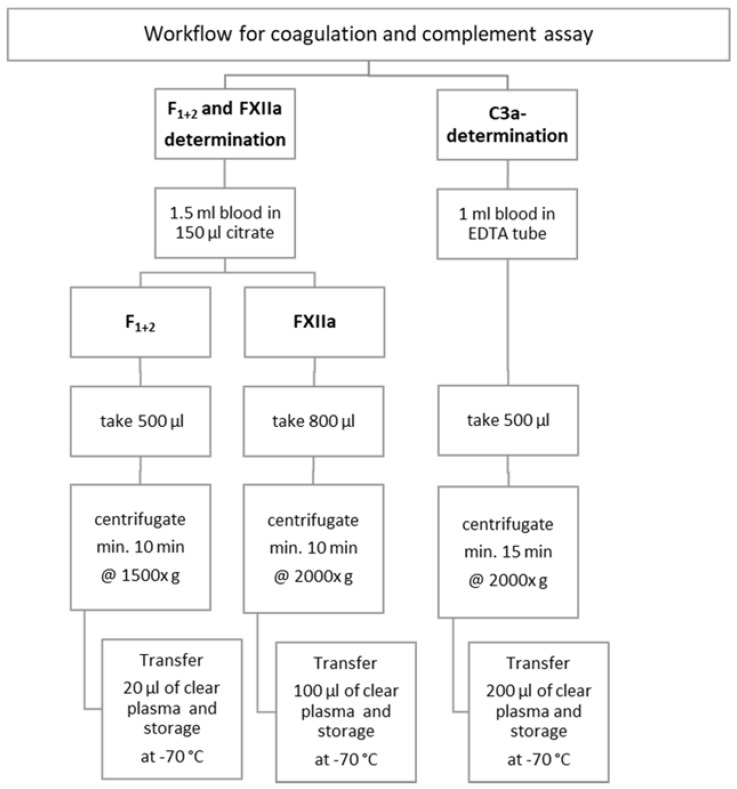
Workflow of preparing blood samples for ELISA assays and testing markers of activated coagulation and complement system.

Samples were then prepared for testing the markers of activated coagulation and complement activation according to the workflow shown in Figure 1.

ELISA assay (Enzygnost^®^ F_1+2_ Assay, Dade Behring, Marburg, Germany) was used to measure the prothrombin activation quantitatively. To determine the degree of the activation of the extrinsic haemostasis pathway via Factor FXII activation into FXIIa quantitatively, an “amidolytic substrate assay” was used to measure the Factor XIIa-like activity (UNITEST, Haemochrom Diagnostica, Essen, Germany). Inside prepared human plasma, the FXIIa-like activity is measured by ^α^XIIa bound to α2-macroglobulin using a chromogenic substrate. Photometrical measures determine the release of p-nitroanaline (pNA); with that, the amount of FXIIa-like activity can be calculated. Using the commercially available and reliable complement C3a-desArg-ELISA (PROGEN biotechnology, Heidelberg, Germany), the content of C3a-desArg, as a stable version of the short-lived C3a, is quantified in plasma. This ELISA is based on the H13 antibody, which blocks the short-living C3a, allowing us to determine its quantity in plasma [8]. This gives us an insight into the initial complement activation of novel formulated bone substitute materials.

Contact angle measurements with freshly drawn human blood were conducted to evaluate the material specific wettability in comparison to that of untreated glass. For this purpose, bone substitute beads were crushed to fine powder using a mortar, and dissolved in 3 mL of 70% ethanol and 1 mL H_2_O. To achieve solutions of comparable mass contents or Herafill^®^ G 4 beads were used; for Osteoset^®^, 10 beads were used. Subsequently, the calcium sulfate dispersions were homogenized with a ptfe pestle. Three hundred microliters of the resulting suspension were pipetted on glass slides and dried to achieve homogenous surface layers of the examined bone substitute materials. Ten microliters of freshly drawn blood were pipetted onto relevant test surfaces with a Herafill^®^-G or Osteoset^®^ layer; as a comparison control surface, untreated glass was used. The contact angles were determined on eight independent samples per type of material by means of magnified photographs; the software ImageJ v1.47 (ImageJ, U. S. National Institutes of Health, Bethesda, MD, USA), in combination with the DropSnake plugin, facilitated contact angle measurements.

## 3. Results

**Figure 2:**
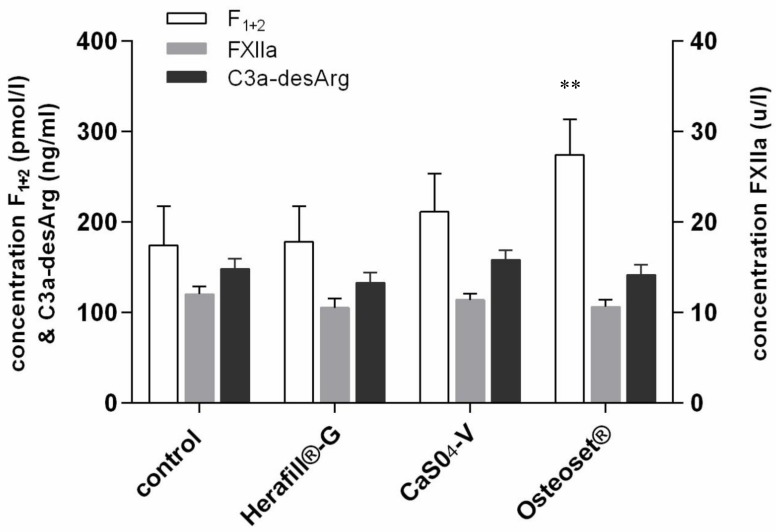
Coagulation markers following incubation of blood with tested beads (*n* = 16). Levels of significance used were **: *p* < 0.01.

Fragment F_1+2_ is a peptide, being split from inactive prothrombin during coagulation, forming the active thrombin. The amount of thrombin is proportional to the amount of F_1+2_, allowing us to quantify the coagulation process. Figure 2 displays the F_1+2_–concentration, as well as the FXIIa and C3a-desArg, after 8 min of incubation of human blood with the bone substitute formulations, as well as the control groups consisting of untreated blood samples. The reference range for F_1+2_ concentration was between 69 and 229 pmol/L. Reference values are below 150 ng/L plasma EDTA; values are regarded as elevated if above 200 ng/L plasma.

The Osteoset^®^ group showed the highest value of F_1+2_ activation; at 274.3 pmol/L, it was highly significant (*p* < 0.01; **), compared to pure blood activation and outside the assay’s reference range. Both tested calcium sulphate formulations containing tripalmitin (Herafill^®^-G and CaS0_4_-V) remained at 178.0 and 211.1 pmol/L, i.e., within the range of reference.

Activation of factor XII in the endogenous coagulation cascade leads, via the shedding of fragment F_1+2_, to a development of prothrombin into active thrombin, catalyzing the formation of fibrin. The reference range is between 14 and 27 u/L; all tested substances remained at a range of between 10.5 and 12.2 u/L, i.e., below the reference range, and thus, without any influence on this coagulation factor.

The complement C3a-desArg ELISA quantifies the content of C3a-desArg as a stable form of short lived C3a in plasma. The results showed values between 132.8 and 158.0 ng/mL, i.e., all remaining below the upper reference level of 200 ng/mL.

**Figure 3:**
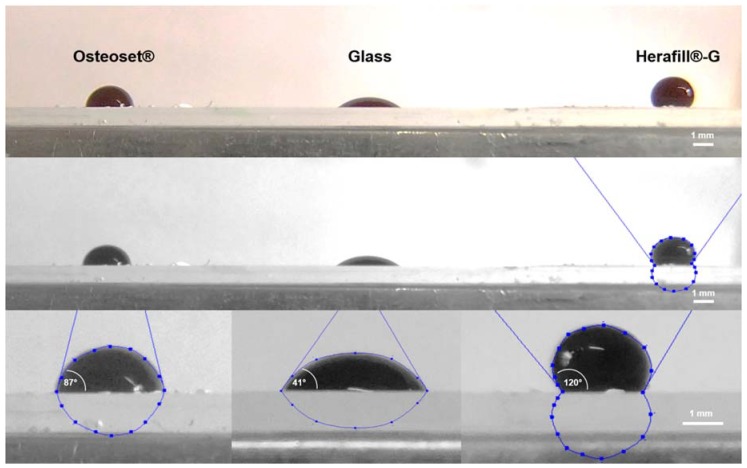
Contact angle measurement in graphic comparison. Magnifications showing average contact angles of fresh human blood on test surfaces (*n* = 8). White scale bar indicating 1 mm.

Contact angle measurements yielded statistically significant results, underlining the different degrees of wettability. Herafill^®^-G displays the lowest degree of wettability (high hydrophobicity), represented by the highest contact angle, followed by Osteoset^®^ (medium hydrophobicity), and the glass as negative control (highly hydrophilic). The specific results are displayed in Table 1 and Figure 3.

## 4. Discussion

Internal use of calcium sulfate (CS), also known as plaster of Paris, has been employed for bone reconstruction for more than a century [1]. While offering high biocompatibility, its influence on coagulation by dissociation has been discussed, but not examined thoroughly to date [9]. A surface-induced coagulation is triggered by factor XIIa, itself having further influence on the complement system, as well as fibrinolysis.

The presented results prove that the tested dihydrate preparations do not accelerate the global hemostasis, nor the tested specific markers of coagulation. Osteoset^®^ samples yielded elevated F_1+2_ concentrations, indicating elevated thrombin formation. Simultaneously, no elevated FXIIa activity is measurable. The observed acceleration of plasmatic coagulation is triggered by the elevated concentration of calcium-ions in the Osteoset^®^ preparation. Addition of hydrophobic tripalmitate renders the originally hydrophilic calcium sulfate formulation more hydrophobic. Thus, no relevant quantities of calcium ions are dissolved into the blood, and coagulation processes are not relevantly influenced. The observed contact angle measurement results clearly underline how the tripalmitate addition increases the hydrophobicity, and thus, reduces wettability. Moreover, this could be a hint for one root cause of delayed resorption of beads, as shown by Pförringer et al. [10].

Wettability and dissolution are closely related, and thus, in the observed setup play an important role in the scaffold dissolving process, since contact angles are good indicators for dissolution transition of solid dispersions [11]. In this context, lipid content plays a significant role, as it does in vital body functions, to purposely influence interaction between liquids and solids [12], as shown in our experiment. Interaction between bone substitute material and the surrounding bone tissue has been the focus of recent research, and deserves further attention [13]. The aqueous interaction has been researched, and can partially be connected to the dissolution examination of the compared material [14].

The presence of blood, as well as hematoma, and their effects on the complex process of bone healing, have been discussed in literature [15]. The effects of the presence and addition of platelet rich plasma (PRP) with released growth factors have been tested and discussed, showing the importance of the presence of a scaffold [16] for bone regeneration. Bone’s healing properties after trauma have been investigated [17], yielding a multitude of influencing and decisive factors in the healing process. Shiu et al. even propose that the bone healing response becomes dysregulated if the blood response, and subsequent formation and properties of a hematoma, are altered [18]. Effects of calcium compositions on osteoinduction and—conduction have been researched and described [10], yet the co-influence of bleeding times remains to be tested.

## 5. Conclusions

Modulation of coagulation may influence bone healing. The underlying research compares the interaction of freshly drawn human blood with two calcium dihydrates and one hemihydrate formulation with differing calcium compositions and antimicrobial contents. The two dihydrates showed no influence, while the hemihydrate significantly prolonged coagulation. This can partially be accounted for by the reduced solubility of calcium through the addition of tripalmitate to the formulation [10]. Further research may focus on the influence of coagulation on the resulting hematoma and subsequent bone healing properties.

## Figures and Tables

**Table 1:** Blood contact angle measurement (*n* = 8) mean results in degrees (± standard deviations) and *p*-values of student’s *t*-test referred to glass (Levels of significance used were ***: *p* < 0.001).

Interfaces	Contact Angle (±SD)	Significance
Glass	41.1 (±9.6)°	
Herafill^®^-G	119.6 (±10.4)°	*p* < 0.001; ***
Osteoset^®^	86.6 (±8.4)°	*p* < 0.001; ***
